# Effect of atorvastatin and hydroxychloroquine combination on blood glucose in alloxan-induced diabetic rats

**DOI:** 10.4103/0253-7613.55213

**Published:** 2009-06

**Authors:** Anil Pareek, P.G. Yeole, C.R. Tenpe, Nitin Chandurkar, Ravikiran Payghan

**Affiliations:** Department of Medical Affairs and Clinical Research, Ipca Laboratories Ltd., 142 AB, Kandivli Industrial Estate, Kandivli (West), Mumbai - 400 067, India; 1 Institute of Pharmaceutical Education and Research, Borgaon (Meghe), Wardha, Maharashtra - 442 001, India

**Keywords:** Antihyperglycemic activity, alloxan, atorvastatin, hydroxychloroquine, plasma glucose

## Abstract

**Objective:**

To evaluate the antihyperglycemic activity of atorvastatin and hydroxychloroquine combination in alloxan-induced diabetic rats.

**Materials and Methods:**

Alloxan induced diabetic Wistar male rats were randomized into six groups of 6 rats each. (Normal rats, diabetic control, atorvastatin (ATV), hydroxychloroquine (HCQ), ATV 5 mg /kg + HCQ 100 mg/kg, and ATV 10 mg/kg + HCQ 200 mg/kg). The rats were treated for 9 days and blood samples were collected at baseline and end of therapy. These samples were analyzed for plasma glucose by autoanalyzer. Changes in body weight, water, food intakes and total protein content were also recorded.

**Results:**

Atorvastatin and hydroxychloroquine alone and in combination reported significant fall in blood glucose level from baseline. Fall in glucose level was significantly more in high dose combination of atorvastatin and hydroxychloroquine (ATV: 10 mg/kg + HCQ: 200 mg/kg) as compared to other study treatment groups (ATV: 17% Vs HCQ: 7% Vs ATV 5mg/kg + HCQ 100mg /kg: 14% Vs ATV 10mg/kg + HCQ 200mg /kg: 21%; p<0.01). ATV and HCQ individually and in combination also improved the body weight loss. The weight gain was significantly more in combination treated rats as compared to positive control group and greater than those who received atorvastatin and hydroxychloroquine alone. Rats treated with the combination also reported significant decrease in food intake and significant increase in total protein.

**Conclusion:**

Increased hypoglycemic effect in combination may be due to potentiation or synergism between HCQ and ATV. Further studies are required to demonstrate clinically significant antidiabetic effect.

## Introduction

Diabetes mellitus is a principal cause of morbidity and mortality in man population.[1] Diabetes currently affects an estimated 15.1 million people in North America, 18.5 million in Europe, 51.4 million in Asia, and just under 1 million in Oceania.[2] It is estimated that globally, the number of diabetic patients will rise from 151 million in the year 2000,[3] to 221 million by the year 2010, and to 300 million by 2025.[4] Atherosclerosis is the most common cause of death in diabetes mellitus. An ideal oral treatment for diabetes would be a drug that not only controls the glucose level but also prevents the development of atherosclerosis and other complications of diabetes. Unfortunately, among the currently available drugs, the choice is very limited. Alloxan monohydrate is known for its selective pancreatic islet beta cell cytotoxicity and has been extensively used to induce diabetes mellitus in animals.

Hydroxychloroquine is an antimalarial drug that also acts as a disease-modifying agent in rheumatoid arthritis and lupus erythematosus.[5] The mechanism by which hydroxychloroquine improves hyperglycemia remains unclear. *In vitro* evidence has shown that chloroquine reduces intracellular insulin degradation; increases intracellular insulin accumulation, slows receptor recycling and stimulates insulin-mediated glucose transport.[6]

In an experimental study, hydroxychloroquine significantly elevated insulin-blood concentration resulting in reduced glucose levels in a concentration dependent fashion in diabetic rats.[7] Clinically, hydroxychloroquine showed improvement in sulphonylurea refractory patients with poorly controlled Type 2 diabetes.[8] In another prospective, multicentric observational study of 4905 adults with rheumatoid arthritis, diabetes was reported in 54 patients who had taken hydroxychloroquine as compared to 171 patients who had never taken hydroxychloroquine. It was thus concluded that among patients with rheumatoid arthritis, use of hydroxychloroquine is associated with a reduced risk of diabetes.[9]

Atorvastatin (ATV) is a selective, competitive inhibitor of HMG-CoA reductase used in patients with diabetes and hypercholesterolemia and has been found to be safe and effective.[10] In previous animal study atorvastatin inhibited increase in plasma glucose level and in clinical studies, patients with type II diabetes mellitus exhibited significant decrease in HbA1c level after treatment with atorvastatin.[11] Based on these evidences the present study was planned to demonstrate that combination of atorvastatin and hydroxychloroquine may give better glycemic control along with improved lipid profile.

The objective of the present study was to evaluate the effect of atorvastatin and hydroxychloroquine combination on blood glucose in alloxan-induced diabetic rats.

## Materials and Methods

### Chemicals

Alloxan monohydrate (Sigma Chemical Company), atorvastatin and hydroxychloroquine (Ipca Laboratories Ltd., Mumbai) were used in the study. All other chemicals were obtained from local sources and were of analytical grade.

### Experimental Animals

Wistar male rats weighing 150–200 g were procured from the Laboratory Animal Resource Section of Institute of Pharmaceutical Education and Research, Wardha, India. The animals were housed in 37cm × 23cm × 16cm polypropylene cages with maximum 3 animals per cage and acclimatized for a period of 7 days. Individual animal was identified by a mark on tail with permanent marker and cages were identified with label pasted on cages with relevant information. Animals were housed at a temperature of 24 ± 2°C and relative humidity of 30 to 70%. A 12:12 hr light: dark cycle was followed. All animals had free access to water and standard pelleted laboratory animal diet. The male animals were selected for the studies since the females are shown to be protected from lipid-induced reductions in insulin actions.[12] The experimental protocol was approved by the Institutional Animal Ethics Committee.

### Preparation and administration of drugs

The ATV and HCQ were suspended in vehicle (0.1% w/v suspension of Tween 80 and carboxymethylcellulose (CMC) in water). Animals were deprived of food for 2 hr before dosing. ATV (10 mg/kg) and HCQ (200 mg/kg) were administered in a single dose orally by gavage using a syringe fitted with suitable sized canula. Actual amount was decided on body weight basis. Volume of formulation administered was 0.2 ml per 150 g body weight. After administration animals were fasted for 1 hr.

### Induction of experimental diabetes

Experimental diabetes was induced in rats by injecting alloxan monohydrate intraperitoneally at a dose of 90 mg/kg body weight[13] Alloxan was dissolved in citrate buffer at pH 4.5 and injected immediately within few minutes to avoid degradation. After 72 hr, blood was collected from the retro-orbital plexus by chloroform anaesthesia of all surviving rats and blood glucose levels were determined using autoanalyser (Microlab 2000). Rats with blood sugar level of 200-350 mg/dl, as well as with polydipsia, polyuria, and polyphagia were considered as diabetic and were employed in the study.

### Experimental Design

The rats were randomly divided into six groups consisting of six rats each. Group 1 (normal control) consisted of normal rats that neither received alloxan monohydrate nor any drug. Group 2 served as positive control (diabetic control). Rats in Group 3 were diabetic and treated with atorvastatin (10 mg/kg;p.o.). Rats in Group 4 were diabetic and treated with hydroxychloroquine (200 mg/kg;). Animals in Group 5 were diabetic and treated with combination of low dose atorvastatin (5mg/kg; p.o.) and hydroxychloroquine (100mg/kg;p.o.), whereas diabetic rats in Group 6 were treated with combination of atorvastatin (10mg/kg p.o.) and hydroxychloroquine (200mg/kg;p.o.). The drugs were given once daily for 9 days.[14]

### Estimation of plasma glucose and total protein

Blood samples were collected from 18 hr fasted rats at 1 hr after the last dose administration from the retro-orbital plexus under chloroform anesthesia and analyzed for plasma glucose by autoanalyzer (Microlab 2000). Total protein content was also estimated by using autoanalyzer (Microlab 2000). In addition, the changes in body weight, water and food intakes and total protein content were recorded in both the control and treated groups.

### Statistical analyses

The results were expressed as mean ± SD. All the data were analyzed by one way analysis of variance followed by Dunnett's t-test. A value of *P* < 0.05 was considered as statistically significant.

## Results

### Effects on body weight, water and food intakes

The effect of ATV and HCQ individually and in combination on body weight, food and water intake were assessed for nine days. Alloxan-induced diabetic rats showed marked decrease in body weight. There was considerable increase in water and food intake. ATV (10 mg/kg; p.o.) with HCQ (200 mg/kg; p.o.) significantly (*P*<0.01) improved the body weight as compared to diabetic control [Table 1]. There was no significant (*P*>0.05) change in water intake in normal, diabetic and diabetic treated rats. Diabetic rats showed significant increase in food intake as compared to normal rats. ATV (10 mg/kg; p.o.) and HCQ (200 mg/kg; p.o.) individually showed non significant decrease in food intake (*P*>0.05). However, the combination of ATV and HCQ showed significant decrease in food intake at both doses.

**Table 1:** Effects of ATV and HCQ on body weight, water and food intake in alloxan-induced diabetic rats

*Group*	*Treatment (mg/kg; p.o.)*	*Body weight (g)*	*Water intake (ml/rat/day)*	*Food intake (g/rat/day)*
I. Normal	Untreated	170 ± 15	182 ± 22	11 ± 0.8
II. Control	Vehicle orally	150 ± 12*	184 ± 18ns	13 ± 1.0**
III. ATV	10	165±13ns	172 ± 16ns	12 ± 0.7 ns
IV. HCQ	200	160 ±14ns	180 ± 20ns	12 ± 0.6 ns
V. ATV + HCQ	5 + 100	172 ±18ns	165 ± 22ns	11 ± 0.6**
VI. ATV + HCQ	10 + 200	182 ± 20**	164 ± 15ns	11 ± 1.0**

Values are expressed as Mean ± S.D. (n= 6).

* *P*<0.05;

** *P*<0.01; ns *P*>0.05 (non-significant). Result of Group I was compared with Group II and Group II results with Group III, IV, V and VI. ATV- Atorvastatin, HCQ- Hydroxychloroquine

### Effect on plasma glucose level

Table 2 shows the result of blood glucose values in alloxan induced diabetic rats after a daily treatment with ATV and HCQ individually and in combination for nine days. ATV, at a dose of 10 mg/kg, showed significant decrease in the blood glucose levels of 17%, whereas HCQ at a dose of 200 mg/kg showed 7% decrease in blood glucose level. In combination, ATV (5 mg/kg) and HCQ (100 mg/kg) showed 14 % reduction in blood glucose level. The combination of ATV (10 mg/kg) and HCQ (200 mg/kg) exhibited the maximum (21%) reduction in glucose level.

**Table 2:** Effects of ATV and HCQ on plasma glucose level in alloxan-induced diabetic rats

*Group*	*Treatment (mg/kg; p.o.)*	*Plasma Glucose (mg/ dl)*		*% change from baseline*
		
		*Initial*	*Final*	
I. Normal	Untreated	082.1 ± 5.0	084.3 ± 7.2	+03
II. Control	Vehicle orally	305.2 ± 24.7	316.4 ± 23.6**	+04
III. ATV	10	290.4 ± 20.2	239.8 ± 28.4**	−17
IV. HCQ	200	302.3 ± 24.5	282.2 ± 21.3*	−07
V. ATV + HCQ	5 + 100	296.6 ± 18.1	255.2 ± 19.4**	−14
VI. ATV + HCQ	10 + 200	293.3 ± 20.8	231.7 ± 14.0**	−21

Values are expressed as Mean ± S.D. (n= 6). Statistical analysis was carried out by one way analysis of variance (ANOVA) followed by Students t-test.

* *P*<0.05;

** *P*<0.01: Result of Group I was compared with Group II and Group II results with Group III, IV, V and VI. ATV- Atorvastatin, HCQ- Hydroxychloroquine

### Effect on plasma protein level

Table 3 shows the results of plasma protein levels in alloxan induced diabetic rats after a daily treatment with ATV and HCQ individually and in combination for nine days. Administration of ATV (10 mg/kg) and HCQ (200 mg/kg) individually resulted into an increase of total protein in diabetic rats by 5% and 13%, respectively. In combination, ATV (5 mg/kg) and HCQ (100 mg/kg) showed highly significant increase in total protein level by 24% whereas combination of ATV (10mg/kg) and HCQ (200mg/kg) showed the maximum increase in total protein level by 40%.

**Table 3:** Effects of ATV and HCQ on total protein level in alloxan-induced diabetic rats

*Group (S)*	*Treatment (mg/kg; p.o.)*	*Total protein (g/dl)*		*% change from baseline*
		
		*Initial*	*Final*	
I. Normal	Untreated	7.43 ± 0.62	8.85 ± 0.92	+19
II. Control	Vehicle Orally	4.10 ± 0.31	4.45 ± 0.28**	+08
III. ATV	10	4.15 ± 0.46	4.35 ± 0.18 ns	+05
IV. HCQ	200	4.39 ± 0.12	4.96 ± 0.46 ns	+13
V. ATV + HCQ	5 + 100	4.23 ± 0.18	5.26 ± 0.74*	+24
VI. ATV + HCQ	10 + 200	4.29 ± 0.27	6.00 ± 0.68**	+40

Values are expressed as Mean ± S.D. (n= 6). Statistical analysis was carried out by one way analysis of variance (ANOVA) followed by Students t-test.

* *P*<0.05;

** *P*<0.01: ns *P*>0.05 (non-significant). Results of Gr. I was compared with Gr. II and Gr. II results with Gr. III, IV, V and VI. ATV- Atorvastatin, HCQ- Hydroxychloroquine

## Discussion

Diabetes mellitus is a disease characterized by elevated blood glucose levels, resulting from absence or inadequate pancreatic insulin secretion.[15] The prevalence of diabetes is increasing worldwide. Insulin resistance plays a key role in the development of the disease in about 90% of patients with NIDDM. The researchers examined the hydroxychloroquine, a commonly used medication to treat rheumatoid arthritis, for its hypoglycemic effects.[9] In animal models, hydroxychloroquine showed significantly elevated insulin blood concentration resulting in reduced glucose levels in streptozotocin induced diabetic rats.[7]

In addition to hyperglycemia, patients with type 2 diabetes often present with a concomitant atherogenic dyslipidemia (elevated triglycerides, low HDL cholesterol, and elevated LDL cholesterol) that increases risk of cardiovascular disease.[17,18] Because there is a high incidence of mortality for type 2 diabetics with their first myocardial infarction, aggressive therapy for treating diabetic dyslipidemia is recommended.[17]

Atorvastatin (ATV) is a selective, competitive inhibitor of HMG-CoA reductase used in patients with diabetes and hypercholesterolemia and has been found to be safe and effective. In animal model of type II diabetes mellitus, atorvastatin exerts significant lipid lowering effect, glucose lowering effect and decreased plasma insulin levels and insulin resistance index. Atorvastatin may improve insulin resistance.[11]

In the present study, we investigated the antihyperglycemic activity of atorvastatin and hydroxychloroquine when administered alone and in combination in alloxan induced diabetic rats. Both, atorvastatin and hydroxychloroquine when administered alone and in low dose and in high dose combination orally for nine days showed significant decrease in the blood glucose levels. Interestingly, the high dose combination of atorvastatin and hydroxychloroquine exhibited the most significant reduction in glucose level than their low dose combination and when administered alone. Thus, the antihyperglycemic effect reported by hydroxychloroquine in streptozotocin-diabetic rats[7] and atorvastatin in KK/Ay mice[11] was further confirmed in our study.

Alloxan-monohydrate caused body weight reduction that was reversed by combination of atorvastatin (10 mg/kg) and hydroxychloroquine (200 mg/kg) indicating that the combination has beneficial effect in preventing loss of body weight in diabetic rats. [Table 1] The combination has also shown significant decrease in food intake.

Diabetes mellitus is characterized by dysregulation in carbohydrate, fat and protein metabolism. In present study the combination has shown significant increase in the total protein content in alloxan induced diabetic rats. This shows that the combination has improved the protein metabolism.

Along with antihyperglycemic effects, hydroxychloroquine is also associated with cardiovascular benefits. Improvement of serum cholesterol in patients treated with hydroxychloroquine has been reported. These include a decrease in serum cholesterol by approximately 10% and an increase in low-density lipoprotein receptors. Hydroxychloroquine also led to a rise in both HDL and %HDL.[19]

Atorvastatin with hydroxychloroquine can offer a new and promising approach in the management of diabetes mellitus, due to its multifaceted action. Since the combination can produce a better glycemic control along with improvement in the lipid profile in animals, it is worthwhile to evaluate the combination of atorvastatin and hydroxychloroquine at different doses so as to find out the potential of this combination as an antidiabetic therapy. Well controlled clinical studies are required to demonstrate this anti diabetic effect.

## Conclusion

The present study demonstrates that combination of hydroxychloroquine with atorvastatin at the dose level tested exhibits significant glucose lowering activity in alloxan induced diabetic rats. Increased effect in combination may be due to potentiation or synergism.
